# Parents’ Preferences for Sex of Children in Sweden: Attitudes and Outcomes

**DOI:** 10.1007/s11113-018-9462-8

**Published:** 2018-03-03

**Authors:** Vitor Miranda, Johan Dahlberg, Gunnar Andersson

**Affiliations:** 1 Department of Population and Welfare, Statistics Sweden, Stockholm, Sweden; 2 Stockholm University Demography Unit, Stockholm, Sweden

**Keywords:** Gender preference, Family formation, Birth rates, Sweden

## Abstract

It has been argued that preferences for the sex of children would be small or non‐existing in relatively gender equal societies. However, previous studies have suggested that a stronger preference for having daughter exists in Scandinavian countries, which are frequently noted for being among the most gender equal societies in the world. Combining new register data on birth rates by sex of the previous children and recent survey data on couples’ stated preferences for the sex of children, we show that the preference for daughters has increased in Sweden over the last decade. In addition to the stronger preference for having daughters among two‐child mothers documented in previous research, our findings show that during the previous decade this preference was noticeable also among one‐child parents. Despite Swedish society being known for holding gender equal social norms, interviewed parents openly expressed some degree of preference for having daughters over sons.

## Introduction

The issue of parents’ preferences for sex of children has gained increased attention in demographic research. Previously, developing countries were the main focus of research (e.g., Arnold and Kuo 1984; Basu and Das Gupta 2001), but over the past decades there has been an increasing interest in sex preferences in developed countries as well (Hank and Kohler 2000; Marleau and Saucier 2002; Mills and Begall 2010; Tian and Morgan 2015). It is sometimes argued that sex preferences would be small or non-existing in relatively gender equal societies (Pollard and Morgan 2002). However, Andersson et al. (2006) have demonstrated that this assumption does not hold for Scandinavian countries, which are often seen as frontrunners in terms of gender equality policies (Plantenga et al. 2009). Those scholars have shown that the fertility of two-child mothers by the sex composition of their previous children reveals a stronger desire to have a daughter than to have a son in Sweden, Denmark, and Norway, but not in Finland (see also Andersson et al. 2007; Saarela and Finnäs 2014). Their study shows that these patterns emerged in the 1980s and became even more pronounced in the 1990s.

The phenomenon of sex preference for sex of children in a society can be studied from two different angles. While one approach is to make inferences based on the study of observed behavior, such as differential birth rates, another approach is to investigate preferences and attitudes directly reported by parents. In this context, the purpose of the present study is twofold. First, we extend the existing analyses by Andersson et al. (2006) of the birth rates of Swedish one- and two-child mothers by the sex composition of their previous children. We add newly available register data for another decade of observation (2000–2012) to examine whether the distinct pattern of sex preferences for daughters observed in the 1990s has persisted, vanished, or intensified during the 2000s.

Second, we use data from the recently released 2012 Swedish Generations and Gender Survey (GGS) in order to investigate the relationship between parents’ stated and revealed preferences for the sex of a possible additional offspring. The Swedish GGS contains questions on whether interviewees would like to have another child and their preferences for the gender of that possible next child. In addition to providing a clearer picture of parents’ gender preferences for children in Sweden, these data make it possible to evaluate whether parent’s self-reported preferences for the sex composition of their offspring can be matched to the implicit preference for having daughters that we observe in the Swedish register data. The survey data also allow us to study the influence of different factors that may relate to fertility decisions, such as employment prospects, romantic life, and social pressure from family and friends.

The next section includes a brief overview of empirical work that documents preferences for the sex of children in different parts of the world, and the Nordic countries, in particular. This is followed by a presentation of theoretical arguments from the literature to explain why parents might prefer to have sons or daughters and how those preferences might change over time as a country moves towards a more gender egalitarian society. We then present our research questions and provide an overview of our data and methods, followed by the empirical results and a concluding discussion.

## Sex Preferences and Childbearing

Previous studies have demonstrated that parents often have preferences about the sex composition of their offspring, in some cases even adjusting their reproductive behavior to match those preferences. The part of this line of research that has received most attention is perhaps the case of son preferences in societies in South and East Asia. A series of outcomes related to childbearing have been used to show this pattern. Elevated sex ratios at birth (i.e., the proportion of boys to girls in live births) have been found in some countries in the region, including China, Vietnam, India, and the post-Soviet countries of the Caucasus (UNFPA 2012). This pattern has increased in parallel with the wider availability of technologies to determine the sex of the fetus, such as ultrasonography, and has therefore been linked to the practice of sex-selective abortion (Yi et al. 1993).

Another strategy to indicate the prevalence of son preference is to compare the childbearing intentions and behavior among parents with different numbers of sons and daughters. The underlying idea is that, when there is strong preference for sons, couples that have only daughters are more likely to reveal a desire to have an additional child than are other couples. When contraception is accessible, this desire is likely to translate into lower use of birth control and ultimately higher birth rates among couples with only daughters. This pattern was documented in China in the early 1980s, where couples with only daughters showed much lower rate of contraceptive use than other couples with the same family size (Arnold and Zhaoxiang 1986). Moreover, levels of contraceptive use were equally high among parents with only sons and those with a mix of sons and daughters. This suggests that parents with only sons did not consider it important to have at least one female offspring. Differential stopping behavior in childbearing that indicates the existence of preferences for having sons over daughters has also been documented in Armenia, Azerbaijan, India, Jordan, Pakistan, and Nepal (Clark 2000; Bongaarts 2013).

For most contemporary societies outside of Asia, the analyses of differential childbearing behavior and intentions have not indicated that son preferences are important. Rather, most research suggests that parents often want to have a balanced sex composition with at least one girl and one boy in their offspring. This has been found in many countries in Latin America and Sub-Saharan Africa (Arnold 1997), Europe (Hank and Kohler 2000), as well as in the United States (Pollard and Morgan 2002), Canada (McDougall et al. 1999), and Australia (Kippen et al. 2007). Furthermore, in some countries such as Sweden, Norway, and Denmark, having at least one daughter seems to matter more than having at least one son. Although parents with one son and one daughter have the lowest birth rates among different constellations of two-child parents, Scandinavian parents with two sons have considerably higher birth rates than do parents with two daughters (Andersson et al. 2006; Kolk and Schnettler 2013).

Evidently, the use of differences in birth rates to make inferences about sex preferences for children has some limitations. As noted by Raley and Bianchi (2006), it is possible to interpret associations in the opposite way than what is usually done. For example, parents with two boys might be more likely to progress to a third birth not because they necessarily hope for a daughter, but because they so much enjoy their boy children that they are more likely to want another child. For this reason, inferences based on differences in birth rates are enhanced if confirmed by corresponding responses in attitude surveys. This is the approach we apply in the present study, as shown below.

## Theoretical Explanations for Sex Preferences

Most theoretical attention in relation to sex preferences for children has focused on son preferences. Much theory has focused on how gender roles and their interaction with kinship systems determine unequal abilities for sons and daughters to contribute to the household over the life course (Das Gupta et al. 2003). In many historical settings and in contemporary societies in South and East Asia, sons tend to be perceived as the key source of contribution to the household in the form of paid labor or work in the family farm (e.g., Sandström and Vikström 2015). The contributions of daughters tend to center in providing unpaid domestic work and care to younger and older family members. A higher demand for boys over girls may emerge from the dependency among many families on the economic contribution brought by sons, especially in contexts of lower incomes and restricted social security. This gendered division of labor may be compounded by social norms that regulate kinship systems, especially when these systems are patrilineal and residence is patrilocal. In these cases, daughters may be expected to provide care to their husband’s family after marriage rather than to their own parents. In some contexts, parents might be expected to pay dowries for daughters, and inheritance laws may disproportionally favor male offspring (Basu and Das Gupta 2001).

Since son preferences have been attributed to unequal gender roles in patriarchal societies, it could be expected that sex preference for children would be virtually non-existent in societies with greater gender equality. The empirical evidence on sex preferences and childbearing reviewed above does not fully support this narrative, however, for two main reasons. First, in many societies that have less rigid gender roles and in which there is no clear son preference, a preference for a child of *each sex* is still evident. This is, for example, the case in the United States (Pollard and Morgan 2002), Australia (Kippen et al. 2007) and much of Europe, including Sweden (Hank and Kohler 2000; Andersson et al. 2006). This indicates that in those societies, sons and daughters are not viewed as equivalent values to parents (e.g., Hoffman and Hoffman 1973).

A second empirical finding that problematizes the hypothesis that sex preferences for children would be unimportant in societies with greater gender equality comes from recent analyses of changes in sex preferences over time. While most studies of sex preferences are based on cross-sectional comparisons of countries, mostly due to data limitations, a few studies have been able to produce long and consistent time series for the same country. Some examples are the studies of the Nordic countries by Andersson et al. (2006, 2007), discussed above. In international comparisons, those countries are often placed among those with the highest level of gender equality, and continued progress in that area has been made over the last decades (World Economic Forum 2016; Frejka et al. 2017). Therefore, one may expect that parents in those societies would become more neutral over time to sex preferences and that birth rates would become gradually more independent of the sex composition of previous offspring. However, the analysis of third birth risks by Andersson and colleagues suggested otherwise: having at least one daughter seems more important to parents in contemporary Denmark, Norway, and Sweden than having at least one son. More significantly, this pattern emerged around the 1980s and had not shown any sign of decline until the late 1990s, the last data point available in the literature. In short, the developments in those Nordic countries point to the opposite direction that would be expected by a straightforward association between gender equality and the decline of sex preferences, as entirely new patterns of differential birth rates have emerged in the recent past.

The theory of a two-stage gender revolution, as put forward by Goldscheider et al. (2015), can help organize the discussion around this apparent contradiction between gender equality and persistent preferences for the sex composition of offspring. The theory suggests that recent gains in gender equality and the decline of the male breadwinner family model in developed societies can be analytically divided into two phases, referring to changes in the public and private sphere, respectively. Changes in the public sphere relate to increases in female participation in the previously male-dominated market of paid labor. The second phase relates to increasing equality in the division of labor in the private sphere, by which men participate more actively in unpaid domestic work, such as childrearing. The two stages of the gender revolution tend to follow each other in a temporal manner, but a temporal gap between the two stages may create a “second shift” for women (Goldscheider et al. 2015). This concept refers to the situation when women accumulate the double burden of contributing to paid work and being responsible for household work while the second part of the gender revolution is not complete.

Given the multiple dimensions of gender equality, it is difficult to assess how far the second part of the gender revolution has advanced in Sweden. On the one hand, Swedish men hold an increasing share of domestic tasks. Public policies promote gender equality in the use of parental leave and fathers’ uptake has increased in recent years (Duvander and Johansson 2012; Statistics Sweden 2016). On the other hand, men and women tend to specialize in different types of housework (Kan et al. 2011) and the vast majority of parental leave days continues to be used by mothers (Statistics Sweden 2016). Available statistics on care for elderly parents provide yet additional evidence of the difficult convergence of gender roles in the private sphere. Institutional care and home help services to the elderly were cut back substantially during the 1980s and 1990s and female relatives took most of the extra share of required care. By 2000, daughters in Sweden were two and a half times more likely than sons to be the care provider to an elderly parent who lived alone and had needs (Johansson et al. 2003).

In our study, we use the framework of the two-stage gender revolution to help explain sex preferences for children and the recent developments in birth rate differentials in Sweden. Applied to the study of preferences for the sex of children, a slow progress in the second part of the gender revolution means that parents continue to see sons and daughters as having some inherently different traits and strengths, even if gender roles are apparently more flexible. With the increase in female labor force participation, parents may expect that both sons and daughters can provide financial help at old age, or at least support themselves and their own families, but that daughters might continue to be seen as a more reliable source of care and social support. This narrative is compatible with the desire in Sweden to have at least one daughter.

## Research Questions

Given the different pace of progress in the different dimensions of gender equality, it is difficult to tell in advance the impact those changes may have had on preferences for the sex of children in Sweden during the last decade. To address this issue in more detail, we use newly available register data to examine women’s transitions to a second and third birth, respectively, conditional on the sex of the existing children. This is complemented by the study of responses to a survey on attitudes related to childbearing conducted in Sweden during 2012. In practical terms, our study addresses four main research questions, as follows:

1. Has the transition to a third birth become more neutral to the sex composition of the previous children since 1999?
2. Has the transition to a second birth remained neutral to the sex of the first child since 1999?
3. Do stated preferences regarding the sex of the next child available in the survey data confirm the inferences made based on differential births rates?
4. What dimensions of life seem to have greater impact on parents’ intention to have another child: career and employment; personal and romantic life; or opinions from family and friends? Do those patterns vary by the sex composition of previous children?

Question 1 relates directly to the links between the progress in gender equality in general and the evolvement of sex preferences for children. The goal is to determine if the patterns of sex preferences as evident in third birth rates during the 1980s to 1990s was a phenomenon exclusive for those decades, or if they have persisted into the 2000s. An eventual convergence to a more gender-neutral pattern of birth rates during the 2000s can be regarded as evidence that the second part of the gender revolution is gaining momentum. Question 2 serves as an additional check to that same issue.

Question 3 has both substantive and methodological significance. First, this part of the analysis presents the most up-to-date survey data that are available on parents’ stated preferences for sex of children in Sweden. Second, the results give additional evidence on whether the conclusions inferred from differential birth rates are an adequate interpretation of sex preferences. This is an important methodological contribution, since this issue was not addressed in the studies by Andersson et al. (2006, 2007).

Finally, question 4 makes a step towards a better understanding of some of the mechanisms that may drive preferences for the sex of children. With the available survey items at hand, it is possible to investigate, for instance, if parents of only sons are less worried than other parents about the impact of an additional child on their employment opportunities or other aspects of working and couple life. [These survey items build on theoretical frameworks on reasoned action and planned behavior proposed by Fishbein and Ajzen (1975) and Ajzen and Fishbein (1980)]. Moreover, available questions on the perceived opinions of family and friends on respondents’ childbearing decisions help provide additional insight into decision mechanisms that has been lacking in previous research on sex preferences for children. While much research has focused on individual and institutional characteristics related to fertility intentions, the study of the role of meso-level family and social networks in shaping fertility preferences has received much less attention (Balbo and Mills 2011; Bernardi and Klärner 2014). This holds for fertility research in general and evidently also for research on childbearing decisions in relation to any sex preferences for children.

## Data and Methods

The present study uses Swedish population register data provided by Statistic Sweden (2003) in order to investigate birth rates by parity and the sex of the existing child or children. These register data contain rich information on all individuals that ever lived in Sweden between 1961 and 2012, including dates of birth, death, and international immigration and emigration. Personal identifiers allow us to link parents to their children and to create full birth histories. We focus our analysis on second and third births that took place between 1970 and 2012 by native-born women born in 1925 and onward.

Using event history analysis techniques, we estimate piecewise constant exponential models (Hoem 1993; Blossfeld et al. 2007). The data are organized with monthly precision of exposures and birth outcomes. We use the sex of the previous child(ren), calendar year, age of the mother, and time since the previous birth as independent variables. Those variables are coded as categorical variables. Age of the mother is represented by ten intervals of size 3 years (i.e., from ages 16–18 to ages 43–45). Time since the previous birth is also divided into ten intervals: 0–1, 1–1.5, 1.5–2, 2–2.5, 2.5–3, 3–4, 4–5, 5–6, 6–8, and 8–10 years. Therefore, women are right censored in the study if they do not experience a birth within ten years after their previous birth. Calendar year is coded as dummy variables representing single years. Finally, an interaction term between calendar year and the indicator of the sex of the previous child(ren) reveals the trend in parents’ implicit preferences for the sex of their children over time. In other words, we interpret differences in parity progression rates by sex of existing children as an indication of parents’ preferences for the sex of children. Our results are presented as relative birth risks standardized by the mentioned variables.

To study parents’ attitudes on preferences for the sex of children we use the 2012 Swedish Generations and Gender Survey (GGS) (Thomson et al. 2015). The GGS is part of the Generations and Gender Programme and, as of 2015, it has collected demographic and socioeconomic information on the adult population in nineteen European countries (Vikat et al. 2007; Thomson et al. 2015). In Sweden, the GGS was a telephone-based survey with a target population of 18,000 individuals aged 18–79 years. In total, 9688 responded to the survey (response rate 53.8%). After the initial telephone interview, respondents were also sent a follow-up questionnaire by post or in electronic form and 6830 responded. This study uses data both from the main and follow-up questionnaires.

In particular, our study population consists of one- and two-child parents who were in childbearing ages at the time of the interview. This includes women between ages 18–44, partnered men whose female partner was between ages 18–44 (married or in cohabitation), and single men older than 18. This follows the definition used by the GGS survey, since most questions on fertility preferences were only asked to this subpopulation. A total of 497 one-child and 922 two-child parents reported whether they would like to have another child.^1^ Those who reported a desire to have another child were also asked their preference for the sex of that child. Besides “boy” and “girl,” there was the option “it does not matter.” We also use data from the follow-up questionnaire to look into parents’ attitudes regarding how an additional child might affect their lives in the future and whether they felt pressure from family and friends to have another child. Following the same definition of childbearing age, a total of 512 two-child parents responded to the follow-up questionnaire.

All estimates based on the GGS data used sample weights. Confidence intervals for proportions were calculated using a logit transform, which prevents overshoot in the case of very low or very high proportions—i.e., the boundaries of estimated intervals cannot reach below zero or above 100% (Brown et al. 2001).

## Birth Rates of One- and Two-Child Mothers

**Fig. 1:**
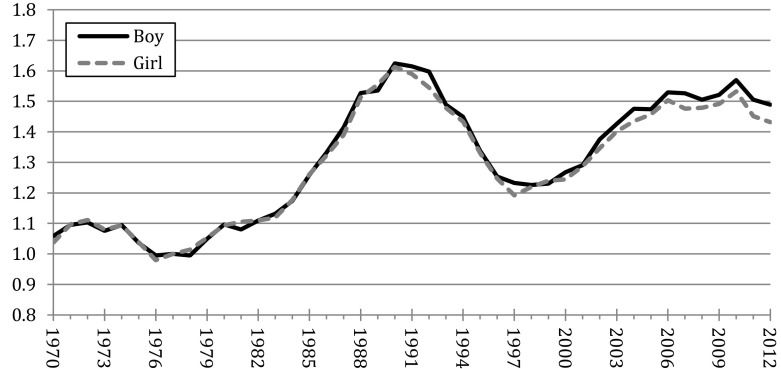
Birth risks of Swedish one-child mothers, by the sex of the first child, 1970–2012 (risks relative to one-daughter mothers in 1977). *Source* Swedish population register, authors’ calculations. The rates are standardized for age of the woman and time since previous birth

Figure 1 shows the register-based relative risk of having a second child among one-child mothers by the sex of their first child. These birth risks are expressed in relation to the birth rate of one-daughter mothers in 1977. The data indicate that between the 1970s and the 1990s there was no substantial difference in the birth rates of one-daughter and one-son mothers. This matches the pattern that would be expected in a society in which parents have strong preferences for having at least two children but no strong preference for the sex composition of their offspring and, therefore, they proceed to second births regardless of the sex of their first child. However, the new data reveal that a new pattern emerged in the first decade of the 2000s, when one-boy mothers started to show slightly higher birth rates than one-girl mothers. For instance, by 2012 the standardized birth rate of one-boy mothers was 4% higher than that of one-daughter mothers—as shown by the estimated relative birth risks of 1.49 and 1.43, respectively (Fig. 1). The pattern observed in the last decade suggests that not having a daughter created a greater incentive to try to have a second child, presumably with the expectation that it could be a girl. In a separate analysis, Kaplan–Meier estimates showed that in the period between 2000 and 2012 this difference in birth risks translated into 84.0% of one-boy mothers having a second child within 10 years from the birth of the first child, compared to 83.3% of one-girl mothers (tabulations available upon request).

**Fig. 2:**
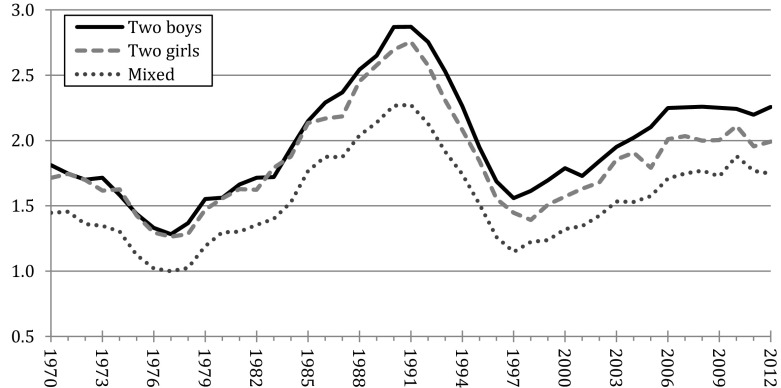
Birth risks of Swedish two-child mothers, by the sex of the first two children, 1970–2012 (risks relative to mothers of mixed-sex offspring in 1977). *Source* Swedish population register, authors’ calculations. The rates are standardized for age of the woman and time since previous birth

Figures for two-child mothers are shown in Fig. 2 and birth risks are expressed in relation to the birth rate of mothers of one boy and one girl in 1977. The results show that throughout the entire period between 1970 and 2012 those mothers who had a daughter and a son consistently showed lower birth rates than the mothers who had either two sons or two daughters. This suggests a relatively stable preference for having at least one child of each sex. Moreover, until the mid-1980s, having two sons or two daughters did not seem to play a significant role in the couples’ decision to have a third child.

Nonetheless, this pattern began to change in the late 1980s and continued to change through the 1990s, when two-son mothers started to show higher birth rates than two-daughter mothers. The new data for 2000–2012 show that this pattern became even more pronounced in more recent years. For instance, in 2012, the standardized birth rate of two-boy mothers was 13% higher than that of two-girl mothers (relative birth risks of 2.26 and 1.99, respectively, as shown in Fig. 2). Kaplan–Meier estimates showed that these different birth rates implied noticeable differences in levels of parity progression. In the period between 2000 and 2012, 36.6% of two-son mothers were estimated to have a third birth within a synthetic follow-up period of ten years, compared to 34.0% of two-daughter mothers, and 30.2% of mothers who had one boy and one girl (tabulations available upon request). Taken together, our results suggest that among the younger generations of Swedish women and their partners the desire to have at least one daughter was clearly stronger than the desire to have at least one boy.

## Parents’ Self-Reported Intentions and Attitudes

The GGS data largely corroborate the overall pattern shown in the register data. In recent years, Swedish parents have been more likely to consider having an additional child if they do not yet have a daughter. The results also help substantiate that this is the consequence of a desire to have at least one daughter. Given the limited sample size of the GGS, even considerable differences between point estimates were at times not statistically significant. Nonetheless, the general patterns found in the data support the interpretation that there is a preference for having at least one daughter in Sweden.

The findings on Table 1 show that the intention to have a second or a third child was higher among parents who had son(s) than among parents who had daughter(s). Sixty-two percent of one-child parents who had a son reported that they would like to have a second child, compared to 59.4% of parents who had only a daughter. The higher desire to have girls was even more apparent among two-child parents. While 23.2% of two-son parents intended to have a third child, 17.7% of two-daughter parents reported so.

**Table 1:** Percentage of Swedish parents who reported an intention to have another child, by the sex composition and number of previous children, 2012. *Source* 2012 Swedish GGS

	%	*p* value for *p*_a_ > *p*_b_	[*N*]
One-child parents			
(a) One-son parents	62.2	0.27	268
(b) One-daughter parents	59.4		229
Two-child parents			
(a) Two-son parents	23.2	0.09	244
(b) Two-daughter parents	17.7		212
(c) Mixed	18.2		466

Women between ages 18 and 44, partnered men whose female partner is between ages 18 and 44, and single men older than 18. Estimates based on the weighted sample

A comparison between the results from the GGS and the register data may suggest that the greater desire to have daughters over sons is more pronounced in the parents’ self-reported intentions than in their actual fertility behavior. Table 1 shows that, in 2012, two-son parents were 1.31 times more likely to report an intention to have a third child than two-daughter parents (i.e., 23.2/17.7). At the same time, Fig. 2 shows that, in that same year, the age-standardized birth rate of two-son mothers was 1.13 times higher than that of two-daughter mothers (i.e., 2.26/1.99). Such comparison should certainly be taken with caution, since the number of observations in the GGS is fairly low. Nonetheless, the difference between intentions and actual behavior might indicate that parents who desire to have at least one daughter sometimes fall short on accomplishing these goals.

**Fig. 3:**
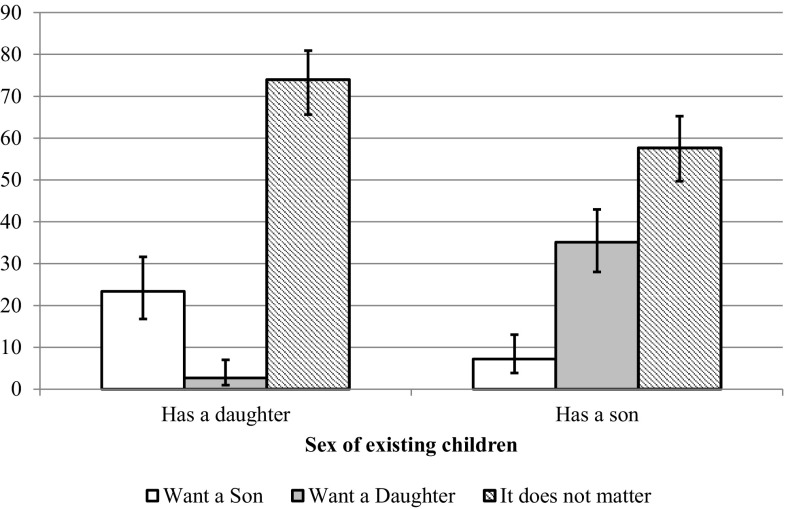
One-child parents’ preference for the sex of their second child, by sex of their first child, Sweden, 2012. *Note* Whiskers show 95% confidence intervals. The sample includes women between ages 18 and 44, partnered men whose female partner is between ages 18 and 44, and single men age 18 or older. Estimates based on the weighted sample. *Source* 2012 Swedish GGS

In the previous section, it was assumed that the higher fertility rates among women who had only sons indirectly revealed a wish that the next child would be a girl. The GGS data allow us to investigate this assumption, since those parents who reported an intention to have an additional child were asked about their preference for the sex of that child. Figure 3 shows that the ideal of gender-neutral preferences is present in the responses of parents in Sweden. Indifference towards the sex of an eventual next child was the most common response among both one-son and one-daughter parents who wanted a second child. However, the results also show that gender neutrality is far from being an absolute norm. Those with a daughter were considerably more likely to say that the sex of the next child would not matter than those with a son (74.0 and 57.7%, respectively). Furthermore, the desire to have at least one daughter is more prevalent than the desire to have at least one son. Over 35% of one-child parents who had a son and wanted another child preferred their second child to be a girl, while only 23.4% of parents who had a daughter preferred their next child to be a boy.

**Fig. 4:**
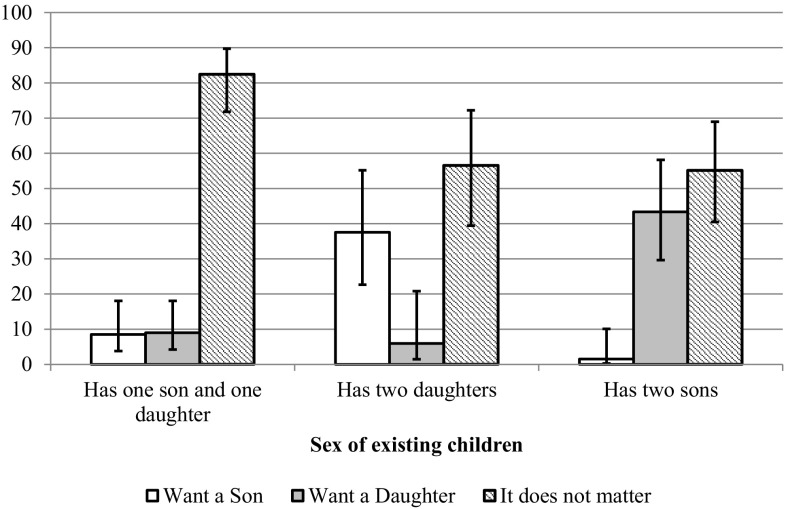
Two-child parents’ preference for the sex of their third child, by the sex composition of their first two children, Sweden, 2012. *Note* Whiskers show 95% confidence intervals. The sample includes women between ages 18 and 44, partnered men whose female partner is between ages 18 and 44, and single men age 18 or older. Estimates based on the weighted sample. *Source* 2012 Swedish GGS

Figure 4 shows preferences for the sex of the next child among two-child parents. Indifference to the sex of an eventual third child was more widespread among those who already had a daughter and a son. Over eighty percent of them said the sex of the next child would not matter. The proportion was 55.1 and 56.5% among two-son and two-daughter parents, respectively. Two-child parents also openly stated their higher desire for at least one daughter compared to at least one son. While 43.3% of parents who had two sons said they preferred their third child to be a girl, only 37.5% of parents who had two daughters expressed a desire to have a boy (Fig. 4).

Finally, we look into a set of questions that inquired how parents suppose that their lives would change if they were to have another child during the 3 years following the survey. The survey also includes questions on whether parents considered that people in their social circles thought they should have another child. Table 2 displays the expressed opinions of two-child parents, by the sex composition of their existing children. Overall, parents expect that their life would become worse in a series of dimensions. Most think that a third child would mean less freedom to do what they want, fewer employment opportunities, a worse financial situation, and worse sexual life, for instance. But, more importantly, these attitudes do not differ consistently by the sex composition of the previous children and none of the observed differences in those dimensions between two-son and two-daughter parents were statistically significant. This suggests that, in general, the higher willingness to have daughters is not associated with a perception that daughters will be easier to raise than boys.

**Table 2:** Attitudes of Swedish two-child parent’s regarding the birth of a third child during the 3 years following the survey, 2012. *Source* 2012 Swedish GGS

	Two sons	Two daughters	Mixed	*p* value of
	(A)	(B)	(C)	A ≠ B
Will… become worse?				
(1) The possibility to do what you want	81.8	85.9	82.9	0.40
(2) Your Employment Opportunities	64.3	70.0	59.4	0.35
(3) Your Financial Situation	76.6	75.1	64.5	0.78
(4) Your Sexual Life	57.9	61.5	46.2	0.58
(5) Closeness with partner	23.6	29.9	26.8	0.27
(6) Partner’s employment opportunities	48.2	48.7	48.7	0.94
(7) Certainty in your life	14.8	20.8	19.1	0.22
Others think you should have another child?				
(8) Most friends	11.2	3.3	9.3	0.02
(9) My parents	12.2	7.3	9.3	0.22
(10) Most relatives	8.1	2.9	8.9	0.08
[*N*]	136	108	268	

Data from the GGS follow-up questionnaire. Estimates based on the weighted sample

The questions on social pressure to have a third child reveal a somewhat different pattern. The vast majority of parents say they do not think their friends or relatives expect them to have another child (lower section of Table 2). But those who have two sons are more likely to report social pressure to have another child than those who have two daughters. Two-son parents were 3.5 times more likely than two-daughter parents to say that most of their friends expected them to have a third child, 1.7 times more likely to feel pressure from parents, and 2.8 times more likely to perceive pressure from other relatives (i.e., 11.2/3.3, 12.2/7.3, 8.1/2.9%, respectively). Pressure from friends was statistically significant at very conservative levels and pressure from relatives other than parents was significant at *p* < 0.10.

Taken together, the findings in Table 2 provide some guidance for future studies. Research that investigates the underlying social mechanisms driving the higher motivation to have daughters in Sweden is advised to not focus solely on how parents perceive the direct benefits of having a girl in their family. One should also consider the pressure that parents notice from their social circles and their willingness to conform to those perceived social norms (cf. Balbo and Mills 2011; Bernardi and Klärner 2014).

## Discussion

Our study was based on register data on actual childbearing behavior and survey data on different subjective dimensions related to childbearing intentions and sex preferences for children. While previous research often reveals a mismatch between childbearing intentions and childbearing behavior (e.g., Toulemon and Testa 2005), we found a correspondence between our data on fertility outcomes and those on parents’ stated preferences for the sex of their next child. In particular, adding information from the 2012 Gender and Generations Survey of Sweden to our study design, we showed that parents’ stated preferences for the sex of their third child matches the pattern of differential birth rates found in the register data.

It has been documented that respondents’ expressed attitudes may be affected by social desirability biases. They might exaggerate socially desirable traits and deny or downplay socially undesirable ones (Tourangeau and Yan 2007; Krumpal 2013). In particular, in Western cultures parents are generally encouraged to treat their children equally (Kowal et al. 2006) and in Sweden gender equality is considered a central norm (Haas 1993; Oláh and Bernhardt 2008). Both women and men in this country express high support for gender equality (Duvander 2014). This narrative would suggest that Swedish parents might be rather unwilling to answer that they have a specific preference for the gender of their next child. Nevertheless, the interviewed parents openly expressed some degree of preference for having daughters over sons.

Our findings demonstrate that the pattern of stronger preferences for daughters than for sons that was observed in Sweden in the 1980s and 1990s has not weakened during the first decade of the 2000s. In contrast, this new pattern has intensified in magnitude and scope. In addition to the clear preference for having daughters among two-child mothers documented by previous research and intensified in the current follow-up, our findings show that during the previous decade this preference was noticeable even among one-child parents. We link our findings to the Swedish context of gender change and the continued progress of the gender revolution in Scandinavia in the spheres of public and private life (cf. Goldscheider et al. 2015). To some extent, the findings mirror those of Mills and Begall (2010) who claimed to find stronger preferences for having sons in low-gender-equity societies in Europe. Contrary to the arguments presented by Pollard and Morgan (2002), the context of increasing gender equality may not produce a situation where parents are indifferent to the gender of their offspring. In contrast, in a society where women are given greater opportunities to develop their potential as actors in the public sphere, daughters may become more valuable for parents in general and for society at large. In such a situation, daughters may be perceived as better than sons in fulfilling both the caring and breadwinning duties that parents may value (Brockmann 2001).

Our further analysis of survey data complements this narrative. We relied on survey items on decision-making processes in relation to the perceived costs and benefits of having another child as well as the possible role of respondents’ social networks in childbearing decisions. In this respect, our study provides support for the notion that parents also weigh in the opinions of their social networks when making decisions about whether or not to have another child. Evidently, such factors merit further attention in different types of fertility research and in theory development in relation to sex preferences for children (Balbo and Mills 2011; Bernardi and Klärner 2014; Hedström and Swedberg 1996). In our case, we provided evidence for the possibility that the perceived pressure from social networks in childbearing decisions may differ based on the sex composition of existing child(ren).

In most developed countries, girls today perform better than boys in educational systems and in many aspects also in society at large. For example, less gender-restricted educational opportunities tend to favor women to develop their cognitive abilities more efficiently than men (Weber et al. 2014). Therefore, whether the development of increasingly strong preferences in Swedish society for having daughters will continue its pace and whether this pattern will become visible also in societies outside Scandinavia remains a topic for future research.^2^ A prediction on more long-term developments in sex preferences for children may still be that such preferences will eventually lose in importance. If the gender revolution as formulated by Goldscheider et al. progresses towards a stage of gender equity in both the private and public spheres of life we may expect that gender may gradually lose importance in many areas where it matters today. However, many factors are at play and it remains an open question whether sex preferences will vanish or not. The evidence provided so far certainly gives no support for predictions of that kind.

We conclude with a note that the emerging patterns in Sweden of preferences for having daughters are manifested in childbearing behavior mainly in the mild version presented in our study. An inspection of differences in sex ratios at birth, by parity and sex composition of previous children, shows no evidence of more drastic interventions in the childbearing process among Swedish-born parents, such as those related to sex-selective abortion (data not shown but available on request).

## Footnotes

1 None of these parents refused to answer this question. Two one-child parents answered “do not know” and were not included in the tabulations. No two-child parent answered “do not know.”

2 Surely, educational performance is just one of the many factors that can affect how parents form preferences for sex of children. Another example is the gender pay gap. Recent data show that the gap has been decreasing steadily since 2007. Women’s salary was 88% of men’s in 2016, compared to 84% in 2007. When variables such as the distribution of men and women into different professions are taken into account, women’s salary was 95% that of men’s in 2016 (Swedish National Mediation Office 2017). Although the gender pay gap has not been eliminated, this growing equality in pay suggests that its role in shaping parents' preferences for sex of children might be declining.
